# Repeat stereotactic body radiotherapy (SBRT) for local recurrence of non-small cell lung cancer and lung metastasis after first SBRT

**DOI:** 10.1186/s13014-018-1080-4

**Published:** 2018-07-28

**Authors:** Yasutaka Ogawa, Yuta Shibamoto, Chisa Hashizume, Takuhito Kondo, Hiromitsu Iwata, Natsuo Tomita, Hiroyuki Ogino

**Affiliations:** 1 Department of Radiology, Nagoya City University Graduate School of Medical Sciences, Nagoya, Japan; 2 Nagoya Radiosurgery Center, Nagoya Kyoritsu Hospital, Nagoya, Japan; 3 Department of Radiation Oncology, Nagoya Proton Therapy Center, Nagoya City West Medical Center, Nagoya, Japan

**Keywords:** Stereotactic radiotherapy, SBRT, Lung, Metastasis, Local recurrence, Retreatment

## Abstract

**Background:**

This study evaluated the safety and efficacy of repeat SBRT for local recurrence of stage I non-small-cell lung cancer (NSCLC) and solitary lung metastasis.

**Methods:**

Thirty-one patients with in-field local relapse of NSCLC (*n* = 23) or lung metastasis (*n* = 8) underwent repeat SBRT. All patients had grade 2 or lower radiation pneumonitis after the first SBRT. Local recurrence was diagnosed with CT and FDG-PET in 17 patients and by biopsy in 14. The median interval between the first and second SBRT was 18 months (range, 4–80). The first SBRT dose was mainly 48–52 Gy in 4 fractions (*n* = 25) according to the institutional protocols. Second SBRT doses were determined based on the tumor size and distance to organs at risk, and were mostly 48–52 Gy in 4 fractions (*n* = 13) or 60 Gy in 8 fractions (*n* = 13).

**Results:**

At 3 years, overall survival and local control rates were 36 and 53%, respectively, for all 31 patients. Four patients showed no further recurrence for > 5 years (63–111 months) after the second SBRT. Radiation pneumonitis after the second SBRT was grade 2 in 4 patients, and no grade 3 pneumonitis was observed.

**Conclusion:**

Repeat SBRT was safe. Local control and survival rates were higher than expected. SBRT should be an important treatment option for local recurrence of NSCLC or lung metastasis after previous local SBRT.

**Trial registration:**

This retrospective study was approved by the ethics committee of our institution (September, 2017; approval number: 27–10).

## Background

Stereotactic body radiotherapy (SBRT) has been established as one of the standard treatments for stage I non-small-cell lung cancer (NSCLC) and solitary lung metastasis. Excellent survival rates have been reported, especially following SBRT for stage I NSCLC, with a high quality of life and a high local control rate of 70–90% [1–3]. In our previous studies, the 5-year local control rate was 83–85% [3, 4]. A recently published report involving combined analysis of two randomized studies of SBRT versus surgery for stage I NSCLC revealed that SBRT yielded better survival rates than surgery [5]. Nevertheless, a proportion of patients develop recurrence or metastasis, and local in-field recurrence remains one of the major patterns of failure.

Treatment of local recurrence of tumors after SBRT has not yet been established. Since many patients were judged inoperable before SBRT, surgery is generally considered difficult, except for in a minority of cases. Chemotherapy may be applied, but cure of the recurrent tumors may be difficult. Traditionally, reirradiation has been considered to be high-risk for most late-responding normal tissues, since damage caused by radiation therapy tends to remain for a long time [6, 7]. However, recent investigations have revealed that re-irradiation could be relatively safely applied to tumors at various sites, especially when the treatment volume could be limited to a small one [8–10]. Previous studies suggested that late radiation damage that was previously considered unrecoverable may recover to some extent [11], and this may especially be the case for the lung [12].

Since SBRT is quite a localized treatment, we hypothesized that retreatment for local recurrence of stage I NSCLC and solitary lung metastases with SBRT using doses similar to those employed in the first-line treatment might be safely delivered. Thus, we used repeat SBRT for such patients. In this report, we describe our results in 31 patients. To our knowledge, our series is the largest one with the longest follow-up periods among studies of second SBRT for lung tumors.

## Methods

### Study design

This was a retrospective analysis of second-line treatment with repeat SBRT for patients who had undergone SBRT for stage I NSCLC and solitary lung metastasis. This study was approved by the institutional review board (No. 27–10). Written informed consent was obtained from all patients. The vast majority of the patients analyzed in this study had entered our previously reported SBRT trials [4, 13, 14]. The SBRT protocols did not define second-line treatment, so we delivered the second SBRT when it was considered to be indicated. In principle, patients with (1) WHO performance status 0–2; (2) no ≥ grade 3 radiation pneumonitis after the first SBRT; (3) arterial oxygen pressure ≥ 60 mmHg; and (4) forced expiratory volume in 1 s ≥ 700 mL were considered eligible. Numbers (1), (3), and (4) had also been included in the eligibility criteria of our previously published SBRT studies [4, 13, 14].

### Patients

Between July 2004 and February 2017, 31 patients with in-field local relapse of NSCLC (*n* = 23) or lung metastasis (*n* = 8) were retreated with SBRT. Local recurrence was diagnosed with the aid of chest diagnostic radiologists using serial CT examinations combined with FDG-PET findings (maximum standardized uptake value [SUVmax] ≥ 5) and/or biopsy. Biopsy was performed in 15 patients, and recurrence was histologically confirmed in 14 of them; in the remaining one patient, biopsy yielded a negative result, but recurrence was radiologically diagnosed based on the subsequent further enlargement of the tumor. At second SBRT, 8 of the 31 patients were considered operable according to our criteria [14], but none of them wished surgery; other 23 patients were considered inoperable. The median patient age at the second SBRT was 77 years (range, 53–90), and 24 were men and 7 were women. The patient and tumor characteristics are summarized in Table 1. The tumor location was classified as either central or peripheral according to the published criteria [15]. No patient had a ultra-central tumor. No patient underwent concurrent systemic therapy.

**Table 1:** Patient and tumor characteristics

Sex	Male/female	24/7
Age (years) ^a^	Median (range)	78 (58–92)
Histology		
Lung cancer	AD/SCC/NSCLC	10/11/2
Metastasis	Lung SCC/lung AD/lung unknown/	2/1/1/
	colorectal CA/breast CA/HCC	2/1/1
T stage (NSCLC)	T1a/T1b/T2a	10/10/3
Location	Peripheral/Central	22/9
Size at 1st SBRT (mm)	Median (range)	22 (10–58)
Size at 2nd SBRT (mm)(major axis)	Median (range)	32 (12–74)
Radiation pneumonitis after first SBRT	Grade 0/1/2	3/26/2

^a^Age at second SBRT. *AD* adenocarcinoma, *SCC* squamous cell carcinoma, *NSCLC* non-small-cell lung cancer, *CA* cancer, *HCC* hepatocellular carcinoma

### SBRT method

Our SBRT method for previously untreated NSCLC and lung metastasis was described in detail previously [13, 14]. Upon treatment planning for second SBRT, plans for the first SBRT were reviewed, and all plans were considered appropriate in terms of the target delineation, immobilization and simulation process, dose calculation, and verification process, except for the possibly insufficient dose prescription that allowed in-field recurrence. Second SBRT was performed following the method employed in the first SBRT, with slight modifications in dose-fractionation schedules. Briefly, the patients were immobilized with the BodyFIX system (Medical Intelligence, Schwabmuenchen, Germany) or a custom-made thermoplastic cast (Hip-Fix, Med-Tec, Orange City, IA, USA). The gross tumor volume was determined with the aid of FDG-PET, and it was equal to the clinical target volume (CTV) due to the use of PET-CT in all cases. The CTV on CT during three phases (under normal breathing, and with breath holding during expiratory and inspiratory phases) was superimposed to represent the internal target volume (ITV). The planning target volume (PTV) margin for the ITV was 5 mm in the lateral and anteroposterior directions and 10 mm in the craniocaudal direction.

As in the first SBRT, second SBRT (and third to fifth in one patient) was delivered by a linear accelerator (CLINAC 23EX, Varian Medical Systems, Palo Alto, CA, USA or Novalis image-guided system, BrainLAB, Feldkirchen, Germany) with 6-MV photons. Three coplanar and 4 noncoplanar static fields were used. According to the first SBRT protocols, most patients had received various doses in 2 or 4 fractions; dose-fractionation schedules at the first SBRT are shown in Table 2. The schedules for the second SBRT are also shown in Table 2; the 2-fraction schedule was no longer used, and 4-fraction schedules were used in 13 patients, 6 fractions in 2, and 8 fractions in 13. Three patients were treated with 10 or 15 fractions. One patient received SBRT 4 times to the same site and once to a neighboring site; this patient is reported in Results in detail. No other patient received SBRT targeting the same site 3 times or more.

**Table 2:** Treatment details

*First SBRT*		
Dose (Gy/fr)	48/4 [105.6]: 50/4 [112.5]: 52/4 [119.6]:	9: 13: 3:
	36/2 [100.8]: 50/10 [75]:	1: 1:
	60/8 [105]: 55/10 [85.3]	3: 1
PTV (cc)	Median (range)	38.5 (7.7–107.6)
PTV D98 (%)	Median (range)	94.0 (78.8–97.0)
PTV D95 (%)	Median (range)	95.0 (80.5–99.0)
PTV Dmedian (%)	Median (range)	100.3 (96.0–103.4)
PTV D2 (%)	Median (range)	104.6 (99.2–112.0)
ITV (cc)	Median (range)	17.9 (1.7–61.3)
ITV Dmedian (%)	Median (range)	100.8 (96.2–103.5)
Lung V20Gy (%)	Median (range)	4.6 (1.6–11.1)
*Second SBRT*		
Dose (Gy/fr)	48/4 [105.6]: 50/4 [112.5]: 52/4 [119.6]:	6: 3: 4:
	54/6 [102.6]: 60/8 [105]: 55/10 [85.3]:	1: 13: 1:
	60/15 [84]: 48/16 [64.2]	2: 1
PTV (cc)	Median (range)	69.8 (10.2–149)
PTV D98 (%)	Median (range)	93.8 (86.8–100.8)
PTV D95 (%)	Median (range)	96.0 (86.0–100)
PTV Dmedian (%)	Median (range)	100.5 (94.8–105.3)
PTV D2 (%)	Median (range)	105.4 (100.2–113)
ITV (cc)	Median (range)	38.4 (1.5–106)
ITV Dmedian (%)	Median (range)	100.6 (97.6–105)
Lung V20Gy (%)	Median (range)	6.5 (2.4–14.5)
Total PTV Dmedian (BED_10_, Gy)	Median (range)	215.0 (139.4–240.6)
Total Dmax (EQD_2_, α/β = 3, Gy)		
Proximal bronchial tree	Median (range)	226.7 (1.7–322.3)
Esophagus	Median (range)	19.4 (0.8–146.8)
Great vessels	Median (range)	111.2 (9.3–317.1)

*PTV* planning target volume, *Dx* dose received by x% of PTV, *V20Gy* volume receiving 20 Gy, *BED*_*10*_ biologically effective dose-10 Gy, *EQD*_*2*_ equivalent dose in 2-Gy fractions. Figures in [] are BED_10_ in Gy

Pencil beam convolution with Batho power law correction was used for dose calculation algorithm until November 2008 for CLINAC 23EX treatment and until January 2011 for Novalis treatment. Thereafter, the analytical anisotropic algorithm was used. The dose was prescribed at the isocenter; it was ensured that 95% of the PTV was covered with at least 80% of the prescribed isocenter dose.

### Evaluation

Follow-up was performed similarly to that after the first SBRT, as described in detail previously [14]. Briefly, chest and upper abdominal CT was performed at 2-month intervals until 6 months, and every 2–4 months thereafter. FDG-PET was performed whenever necessary. Local re-recurrence was diagnosed with serial CT examinations combined with FDG-PET findings (SUVmax ≥ 5) and no patient underwent biopsy for suspected re-recurrence. Pleuritis carcinomatosa unaccompanied by local recurrence was regarded as distant metastasis. Toxicity was evaluated using the Common Terminology Criteria for Adverse Events version 4. Follow-up after 5 years was conducted at the discretion of the attending radiation oncologist. Overall survival (OS), progression-free survival (PFS), and local control rates were calculated from the start of second SBRT using the Kaplan–Meier method. A Fine and Gray competing-risks regression model was used to estimate local control rates, thereby considering patient death as a competing risk. OS, PFS, and local control rates between the patients with a central tumor and those with a peripheral tumor were compared by the log-rank test and incidences of toxicity between the two groups were compared by Fisher exact test.

## Results

### Survival and local control

**Fig. 1:**
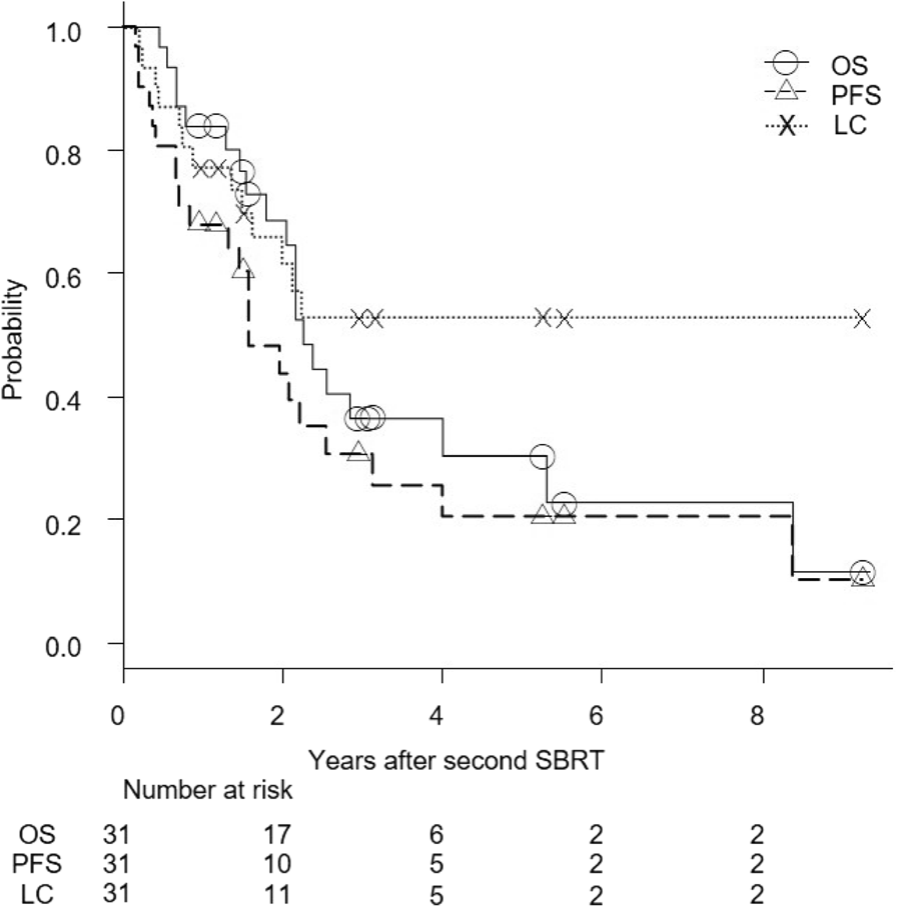
Overall survival (OS), progression-free survival (PFS), and local control (LC) rates after second SBRT in all 31 patients

**Fig. 2:**
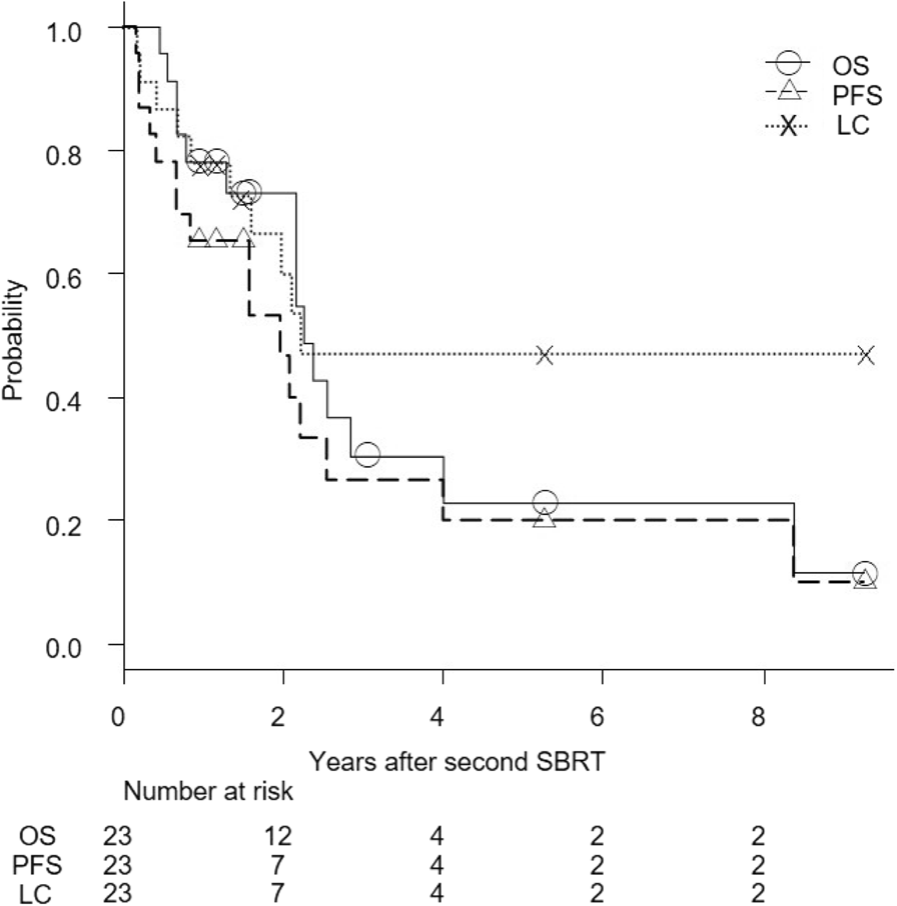
Overall survival (OS), progression-free survival (PFS), and local control (LC) rates after second SBRT in 23 NSCLC patients

The median follow-up period was 26 months (range, 5.5–111 months) for all patients and 35.5 months (range, 11.5–111 months) for living patients. Figure 1 shows OS, PFS, and local control curves for all 31 patients. At 3 years, OS, PFS, and local control rates were 36, 31, and 53%, respectively. For 23 patients with recurrence of NSCLC, these rates were 30, 27, and 48%, respectively (Fig. 2). Five patients, three with NSCLC and one each with metastasis from NSCLC and colon cancer survived for more than 5 years after the second SBRT, four without further local recurrence and one with local recurrence. Two of the three NCSLC patients had a histologically confirmed recurrence of NSCLC. The 3-year OS, PFS, and local control rates were 27, 40, and 40%, respectively, for 6 NSCLC patients with a central tumor, and 31, 25, and 52%, respectively, for 17 NSCLC patients with a peripheral tumor (*P* = 0.75, 0.33, and 0.26, respectively).

### Toxicity

No patient developed grade 3 or higher toxicity after the second SBRT; radiation pneumonitis was grade 0 in 8 patients, grade 1 in 19, and grade 2 in 4 (3/9 in patients with a central tumor and 1/22 in patients with a peripheral tumor; *P* = 0.30). At the last follow-up, 9 patients had rib fractures, but only 3 of them developed fracture after the first SBRT. Skin and soft tissue toxicities were all grade 0 or 1. No other toxicities, including those to the trachea, main bronchus, esophagus, and great vessels, were observed.

### Case presentation

**Fig. 3:**
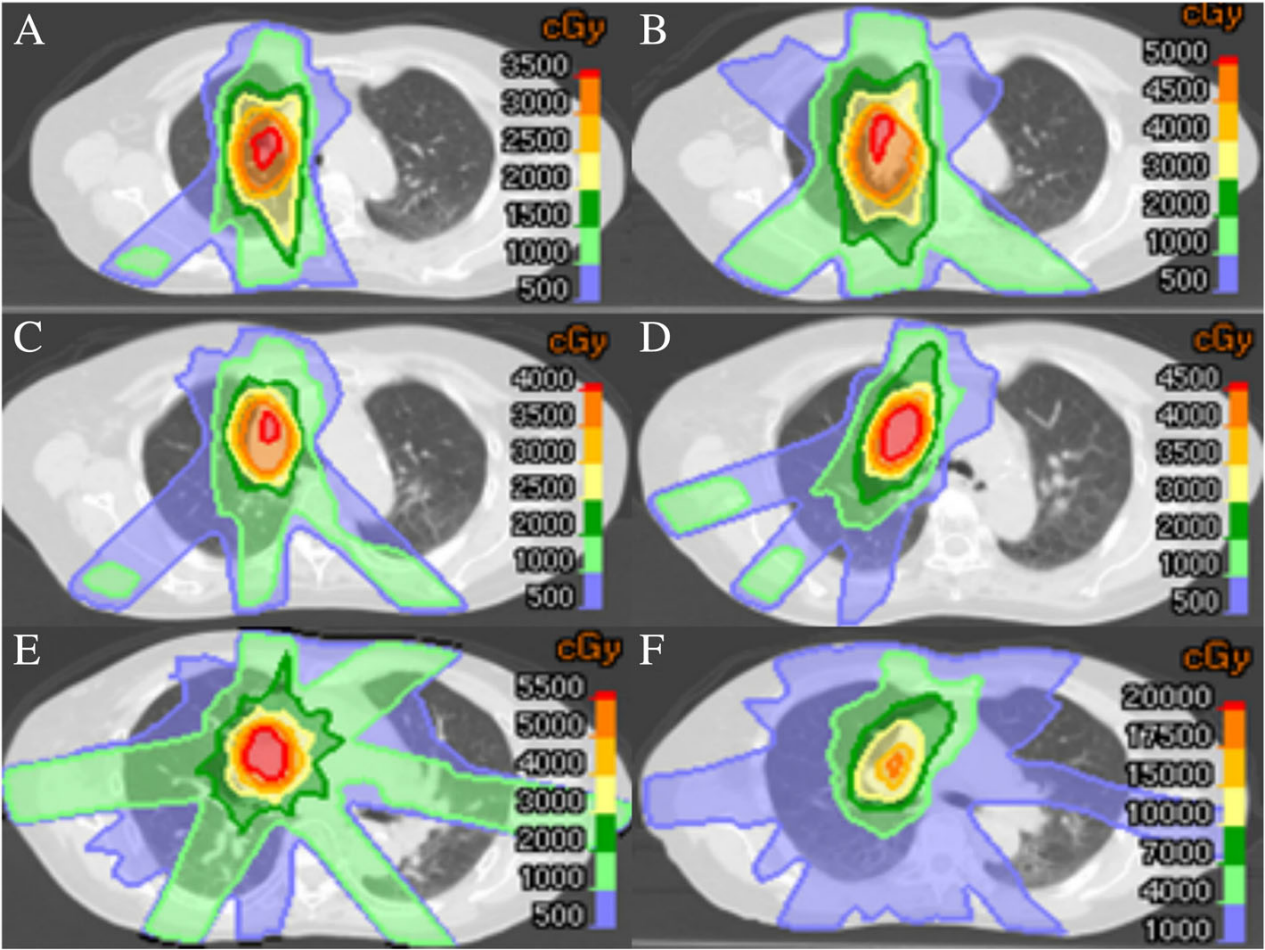
Dose distribution in the first to fifth SBRT for metastatic lung cancer from colon cancer in a 73-year-old female patient. **a**, 1st SBRT; **b**, 2nd SBRT; **c**, 3rd SBRT; **d**, 4th SBRT; **e**, 5th SBRT; **f**, Sum of the 5 treatments

One patient with a solitary lung metastasis from colon cancer underwent SBRT four times to the same site and subsequently once to a neighboring site. Figure 3 shows the dose distributions of these treatments. The prescribed doses at the isocenter for the 5 treatments were 36 Gy in 2 fractions, 50 Gy in 4 fractions, 40 Gy in 4 fractions, 48 Gy in 4 fractions, and 56 Gy in 8 fractions. The intervals between the 5 treatments were 18, 22, 14, and 20 months, respectively. Summing the doses of all treatments, the D_max_ was 134 Gy for the trachea, 141 Gy for the right main bronchus, and 172 Gy for the aorta. The V_20Gy_, V_40Gy_, V_60Gy_, V_80Gy_, and V_100Gy_ of the lung were 33.7, 13.7, 7.0, 3.5, and 1.6%, respectively. This patient only developed grade 1 radiation pneumonitis after the first treatment, and subsequently no toxicity was observed until her death due to brain metastases. After the second to fifth SBRT, the level of carcinoembryonic antigen decreased: from 11.6 to 1.1 by the second SBRT, from 27.8 to 4.7 by the third SBRT, from 141 to 60.3 by the fourth SBRT, and from 604 to 415 by the fifth SBRT (unit: ng/mL; normal range ≤ 5).

## Discussion

Surgery and SBRT are now considered as two major definitive treatments for stage I NSCLC and solitary lung metastasis. So far, the majority of patients undergoing SBRT have been medically inoperable, and so surgery is impossible for patients when they develop local recurrence. On the other hand, numbers of medically operable patients who refuse surgery seem to be increasing, and when they develop local recurrence, surgery may be a second-line treatment option. Successful performance of salvage surgery has been reported [16–18]. Nevertheless, none of the 8 patients who were considered operable desired surgery. Thus, surgery is not indicated for the vast majority of such patients, so repeat SBRT may be a good option to retreat their disease.

Patients retreated with SBRT have been reported in the literature, but the numbers of patients are smaller than ours [19–28]. The previous reports suggested the feasibility and relative safety of second SBRT, and our larger study confirmed the findings. None of our patients developed severe complications. However, three Grade 5 toxicities due to bleeding, one from aorto-esophageal fistula, and one from perforation of gastric ulcer have been reported [19, 22, 29]. The former 4 patients had a central tumor, while the latter one patient had a peripheral tumor. In most of our patients, dose limits recommended in previous SBRT trials for the trachea, major vessels, and esophagus are exceeded when doses for all treatments are summed. In this study, the dose was prescribed to the isocenter, so the delivered dose was lower than with the dose prescription to the isodose line. Nevertheless, recovery from normal tissue damage between SBRT sessions may largely account for the absence of toxicity, and further investigations on the extent of the recovery seem to be warranted. On the other hand, the recommended dose limits would absolutely represent the safe doses below which major complications rarely occur, and dangerous zones may exist at much higher levels. In our patient undergoing 4 sessions of SBRT to the same site and another session to a neighboring site, the Dmax to the trachea, main bronchus, and aorta were > 130 Gy, but no apparent complications developed in these tissues. Our results would suggest relative safety of repeat SBRT; however, considering the potential risk of overdoses, proton beam therapy may be a better option to reduce the doses to the organs at risk.

The efficacy of repeat SBRT was not clarified in previous studies due to the small patient numbers and short follow-up periods. According to the limited previous data, the local control rates at 5 months to 2 years after second SBRT were 50–75% [18–22], but longer-term local control data were not available. Some of the previous investigators employed lower doses at second SBRT, and they might have treated their patients rather palliatively. On the other hand, we used similar doses at both first and second SBRT, and the 3-year OS and local control rates were 36 and 53%, respectively. It should be noted that these rates might possibly be falsely elevated because 55% of our patients had no histological proof of recurrence at second SBRT. However, the present study suggested long-term (> 5 years) tumor control in at least 4 patients. Generally, tumors recurring after radiotherapy may be considered to be radioresistant, since they have not been cured with the first radiation therapy. Therefore, it may be a concern that recurrent tumors cannot be cured with similar treatment. However, this was not necessarily the case in our study. Four tumors were considered cured by second SBRT. Treatment intensities were similar between the first and second SBRT, but the recurrent tumors were slightly smaller than the original tumor in 3 patients and of a similar size in 1 patient. Our study suggests that recurrent tumors are not necessarily more radioresistant, and that patients still have a chance of cure when the recurrence is diagnosed early. Experimental studies with rodent tumors indicated that recurrent tumors were not necessarily more radioresistant and sometimes were more radiosensitive than the original tumors [30, 31].

One of the issues in the retreatment of lung tumors recurring after SBRT is the difficulty in correctly diagnosing recurrence based on imaging studies. It is now well-known that post-radiotherapy changes in the lung may mimic recurrence [32, 33]. Positive FDG-PET findings also do not necessarily indicate tumor recurrence. We used a criterion of SUVmax ≥ 5 to suspect recurrence, but we experienced a number of false-positive cases. Therefore, obtaining a histological diagnosis is desirable. However, bronchoscopic and CT-guided biopsy may yield false-negative results, the case in 1 of our patients. In addition, post-SBRT changes in the lung occasionally involve vascular-rich atelectasis so that CT-guided biopsy may be considered hazardous. This applied to 5 patients in our study. Although repeat SBRT is a relatively safe treatment, delivery of retreatment should be conducted carefully if histological diagnosis cannot be established. Occasionally, enlargement of a tumor-like shadow and a positive FDG-PET finding are not sufficient to diagnose recurrence. If obtaining histology is difficult in such cases, a strategy of watchful waiting may be an option.

## Conclusions

Repeat SBRT appears to be a relatively safe treatment in patients not developing grade 2 or higher radiation pneumonitis after their first SBRT, although grade 5 toxicities have been reported especially in patients with a central tumor. Patients with local recurrence still have a chance of cure by repeat SBRT.

## Abbreviations

CT: Computed tomography
CTV: Clinical target volume
FDG-PET: 18-fluorodeoxy glucose-positron emission tomography
ITV: Internal target volume
NSCLC: Non-small cell lung cancer
OS: Overall survival
PFS: Progression-free survival
PTV: Planning target volume
SBRT: Stereotactic body radiotherapy

## References

[1] Kestin L, Grills I, Guckenberger M, Belderbos J, Hope AJ, Werner-Wasik M (2014). Dose-response relationship with clinical outcome for lung stereotactic body radiotherapy (SBRT) delivered via online image guidance. Radiother Oncol.

[2] Bi N, Shedden K, Zheng X, Kong FS (2016). Comparison of the effectiveness of radiofrequency ablation with stereotactic body radiation therapy in inoperable stage I non-small cell lung cancer: a systemic review and pooled analysis. Int J Radiat Oncol Biol Phys.

[3] Shibamoto Y, Hashizume C, Baba F, Ayakawa S, Miyakawa A, Murai T (2015). Stereotactic body radiotherapy using a radiobiology-based regimen for stage I non-small-cell lung cancer: five-year mature results. J Thorac Oncol.

[4] Miyakawa A, Shibamoto Y, Baba F, Manabe Y, Murai T, Sugie C (2017). Stereotactic body radiotherapy for stage I non-small-cell lung cancer using higher doses for larger tumors: results of the second study. Radiat Oncol.

[5] Chang JY, Senan S, Paul MA, Mehran RJ, Louie AV, Balter P (2015). Stereotactic ablative radiotherapy versus lobectomy for operable stage I non-small-cell lung cancer: a pooled analysis of two randomised trials. Lancet Oncol.

[6] Robbins ME, Bywaters T, Rezvani M, Golding SJ, Hopewell JW (1991). Residual radiation-induced damage to the kidney of the pig as assayed by retreatment. Int J Radiat Biol.

[7] Stewart FA, Oussoren Y, Luts A (1990). Long-term recovery and reirradiation tolerance of mouse bladder. Int J Radiat Oncol Biol Phys.

[8] Murray LJ, Lilley J, Hawkins MA, Henry AM, Dickinson P, Sebag-Montefiore D (2017). Pelvic re-irradiation using stereotactic ablative radiotherapy (SABR): a systematic review. Radiother Oncol.

[9] Kim YS (2017). Reirradiation of head and neck cancer in the era of intensity-modulated radiotherapy: patient selection, practical aspects, and current evidence. Radiat Oncol J.

[10] Shibamoto Y, Yamashita J, Takahashi M, Abe M (1994). Intraoperative radiation therapy for brain tumors with emphasis on retreatment for recurrence following full-dose external beam irradiation. Am J Clin Oncol.

[11] Ang KK, Price RE, Stephens LC, Jiang GL, Feng Y, Schultheiss TE (1993). The tolerance of primate spinal cord to re-irradiation. Int J Radiat Oncol Biol Phys.

[12] Nieder C, Milas L, Ang KK (2000). Tissue tolerance to reirradiation. Semin Radiat Oncol.

[13] Baba F, Shibamoto Y, Tomita N, Ikeya-Hashizume C, Oda K, Ayakawa S (2009). Stereotactic body radiotherapy for stage I lung cancer and small lung metastasis: evaluation of an immobilization system for suppression of respiratory tumor movement and preliminary results. Radiat Oncol.

[14] Shibamoto Y, Hashizume C, Baba F, Ayakawa S, Manabe Y, Nagai A (2012). Stereotactic body radiotherapy using a radiobiology-based regimen for stage I nonsmall cell lung cancer. A multicenter study. Cancer.

[15] Chaudhuri AA, Tang C, Binkley MS, Jin M, Wynne JF, von Eyben R (2015). Stereotactic ablative radiotherapy (SABR) for treatment of central and ultra-central lung tumors. Lung Cancer.

[16] Hamaji M, Chen-Yoshikawa TF, Matsuo Y, Motoyama H, Hijiya K, Menju T (2017). Salvage video-assisted thoracoscopic lobectomy for isolated local relapse after stereotactic body radiotherapy for early stage non-small cell lung cancer: technical aspects and perioperative management. J Vis Surg.

[17] Verstegen NE, Maat APWM, Lagerwaard FJ, Paul MA, Versteegh MI, Joosten JJ (2016). Salvage surgery for local failures after stereotactic ablative radiotherapy for early stage non-small cell lung cancer. Radiat Oncol.

[18] Antonoff MB, Correa AM, Sepesi B, Nguyen QN, Walsh GL, Swisher SG (2017). Salvage pulmonary resection after stereotactic body radiotherapy: a feasible and safe option for local failure in selected patients. J Thorac Cardiovasc Surg.

[19] Peulen H, Karlsson K, Lindberg K, Tullgren O, Baumann P, Lax I (2011). Toxicity after reirradiation of pulmonary tumours with stereotactic body radiotherapy. Radiother Oncol.

[20] Patel NR, Lanciano R, Sura K, Yang J, Lamond J, Feng J (2015). Stereotactic body radiotherapy for re-irradiation of lung cancer recurrence with lower biological effective doses. J Radiat Oncol.

[21] Valakh V, Miyamoto C, Micaily B, Chan P, Neicu T, Li S (2013). Repeat stereotactic body radiation therapy for patients with pulmonary malignancies who had previously received SBRT to the same or an adjacent tumor site. J Cancer Res Ther.

[22] Kilburn JM, Kuremsky JG, Blackstock AW, Munley MT, Kearns WT, Hinson WH (2014). Thoracic re-irradiation using stereotactic body radiotherapy (SBRT) techniques as first or second course of treatment. Radiother Oncol.

[23] Meijneke TR, Petit SF, Wentzler D, Hoogeman M, Nuyttens JJ (2013). Reirradiation and stereotactic radiotherapy for tumors in the lung: dose summation and toxicity. Radiother Oncol.

[24] Ester EC, Jones DA, Vernon MR, Yuan J, Weaver RD, Shanley RM (2013). Lung reirradiation with stereotactic body radiotherapy (SBRT). J Radiosurg SBRT.

[25] Nishimura S, Takeda A, Sanuki N, Yoshida S, Shigematsu N (2015). Dose-escalated stereotactic body radiotherapy (SBRT) as a salvage treatment for two cases with relapsed peripheral lung cancer after initial SBRT. J Thorac Oncol.

[26] Trakul N, Harris JP, Le Q-T, Hara WY, Maxim PG, Loo BW (2012). Stereotactic ablative radiotherapy for reirradiation of locally recurrent lung tumors. J Thorac Oncol.

[27] Hearn JWD, Videtic GMM, Djemil T, Stephans KL (2014). Salvage stereotactic body radiation therapy (SBRT) for local failure after primary lung SBRT. Int J Radiat Oncol Biol Phys.

[28] Caivano D, Valeriani M, De Matteis S, Bonome P, Russo I, De Sanctis V (2018). Re-irradiation in lung disease by SBRT: a retrospective, single institutional study. Radiat Oncol.

[29] Nonaka H, Onishi H, Ozaki M, Kuriyama K, Komiyama T, Saito R (2017). Serious gastric perforation after second stereotactic body radiotherapy for peripheral lung cancer that recurred after initial stereotactic body radiotherapy: a case report. J Med Case Rep.

[30] van Rotterdam A, Barendsen GW, Gaiser JF (1987). Radiosensitivity of cells in recurrent experimental tumours and the effectiveness of tumour retreatment. Radiother Oncol.

[31] Ando K, Koike S, Shikita M, Hayata I, Otsu H, Satoh S (1988). Radiosensitivity of late recurrences following radiotherapy of murine fibrosarcomas. Radiat Res.

[32] Takeda T, Takeda A, Kunieda E, Ishizaka A, Takemasa K, Shimada K (2004). Radiation injury after hypofractionated stereotactic radiotherapy for peripheral small lung tumors: serial changes on CT. Am J Roentgenol.

[33] Takenaka R, Shibamoto Y, Miyakawa A, Hashizume C, Baba F (2016). The fate of residual tumor masses that persist after stereotactic body radiotherapy for solitary lung nodules: will they recur?. Clin Lung Cancer.

